# Preferences for transitional HIV care among people living with HIV recently released from prison in Zambia: a discrete choice experiment

**DOI:** 10.1002/jia2.25805

**Published:** 2021-10-14

**Authors:** Jan Ostermann, Valerie Yelverton, Helene J. Smith, Mirriam Nanyangwe, Lillian Kashela, Peter Chisenga, Vivien Mai, Chilambwe Mwila, Michael E. Herce

**Affiliations:** ^1^ Department of Health Services Policy and Management Arnold School of Public Health University of South Carolina Columbia South Carolina USA; ^2^ South Carolina SmartState Center for Healthcare Quality University of South Carolina Columbia South Carolina USA; ^3^ Center for Health Policy & Inequalities Research Duke Global Health Institute Duke University Durham North Carolina USA; ^4^ Implementation Science Unit Centre for Infectious Disease Research in Zambia (CIDRZ) Lusaka Zambia; ^5^ School of Public Health & Community Medicine University of New South Wales Sydney New South Wales Australia; ^6^ Dalla Lana School of Public Health University of Toronto Toronto Ontario Canada; ^7^ Institute for Global Health & Infectious Diseases Department of Medicine University of North Carolina School of Medicine Chapel Hill North Carolina USA

**Keywords:** differentiated service delivery, discrete choice experiment, HIV infection, incarcerated people, transitional care, Zambia

## Abstract

**Introduction:**

No studies from sub‐Saharan Africa have attempted to assess HIV service delivery preferences among incarcerated people living with HIV as they transition from prisons to the community (“releasees”). We conducted a discrete choice experiment (DCE) to characterize releasee preferences for transitional HIV care services in Zambia to inform the development of a differentiated service delivery model to promote HIV care continuity for releasees.

**Methods:**

Between January and October 2019, we enrolled a consecutive sample of 101 releasees from a larger cohort prospectively following 296 releasees from five prisons in Zambia. We administered a DCE eliciting preferences for 12 systematically designed choice scenarios, each presenting three hypothetical transitional care options. Options combined six attributes: (1) clinic type for post‐release HIV care; (2) client focus of healthcare workers; (3) transitional care model type; (4) characteristics of transitional care provider; (5) type of transitional care support; and (6) HIV status disclosure support. We analysed DCE choice data using a mixed logit model, with coefficients describing participants’ average (“mean”) preferences for each option compared to the standard of care and their distributions describing preference variation across participants.

**Results:**

Most DCE participants were male (*n* = 84, 83.2%) and had completed primary school (*n* = 54, 53.5%), with 29 (28.7%) unemployed at follow‐up. Participants had spent an average of 8.2 months in the community prior to the DCE, with 18 (17.8%) reporting an intervening episode of re‐incarceration. While we observed significant preference variation across participants (*p* < 0.001 for most characteristics), releasees were generally averse to clinics run by community‐based organizations versus government antiretroviral therapy clinics providing post‐release HIV care (mean preference = –0.78, *p* < 0.001). On average, releasees most preferred livelihood support (mean preference = 1.19, *p* < 0.001) and HIV care support (mean preference = 1.00, *p* < 0.001) delivered by support groups involving people living with HIV (mean preference = 1.24, *p* < 0.001).

**Conclusions:**

We identified preferred characteristics of transitional HIV care that can form the basis for differentiated service delivery models for prison releasees. Such models should offer client‐centred care in trusted clinics, provide individualized HIV care support delivered by support groups and/or peer navigators, and strengthen linkages to programs providing livelihood support.

## 1 INTRODUCTION

In sub‐Saharan Africa (SSA) and other high HIV burden regions, prisons concentrate large numbers of people living with HIV (PLHIV), including key populations disproportionately affected by HIV [1, 2, 3]. In Zambia, a land‐locked country in southern Africa, nearly 25,000 people are incarcerated at any time [4]. Published estimates from Zambia indicate that HIV prevalence among incarcerated people is several‐fold higher than among people in the general population, ranging from 20.5% to 27.4% [5, 6, 7, 8]. Mounting evidence from Zambia and SSA suggests that HIV treatment and care can be provided to PLHIV in prisons, resulting in clinical benefits comparable to those seen in the community [9, 10, 11]. Unfortunately, such benefits are short‐lived for PLHIV after release (“releasees”) due to problems with post‐release care continuity posed by multiple psychosocial, health system and structural barriers [6, 12, 13, 14, 15]. Although data to quantify the extent of the problem in SSA are scarce, available reports suggest that one‐third or more of releasees fail to link to community care or experience HIV treatment interruption post‐release [16, 17].

Despite these HIV care disruptions, no studies from SSA have attempted to assess HIV service delivery preferences among incarcerated PLHIV as they transition from prisons into the community, and specific recommendations about interventions to promote post‐release HIV care continuity are lacking [18, 19]. Descriptions of North American transitional care programs highlight service delivery elements that may be applicable to African settings, such as psychosocial support and treatment of co‐morbid substance use disorders [20, 21, 22, 23]. However, client preferences for similar services in SSA are, as of yet, unstudied.

As part of a mixed‐methods prospective cohort study examining longitudinal clinical outcomes among releasees living with HIV [24], we conducted a discrete choice experiment (DCE) to understand releasee preferences for HIV transitional care services in Lusaka, Zambia. A DCE is a quantitative survey method, grounded in random utility theory (RUT) [25, 26] and Lancaster's theory of consumer demand (Lancaster's theory) [27], that can be used to characterize population preferences for goods and services. DCEs have been widely used to elicit preferences for HIV prevention, testing and treatment [28, 29, 30, 31, 32, 33, 34, 35, 36, 37, 38, 39], including in Zambia and elsewhere in SSA [28, 29, 31, 34, 35, 36, 38, 40, 41].

We report here the findings of the first DCE, to our knowledge, conducted with releasees living with HIV in SSA. The aim of this study was to characterize releasee preferences for transitional HIV care services to inform development of a differentiated transitional care model to promote HIV care continuity for this population in Zambia.

## 2 METHODS

This study represents a sub‐study of the Releasee Care Continuum (RCC) study (#NCT02905162). Key elements of this sub‐study are described below; additional details are presented in appendices (Appendix S1). Study activities were approved by institutional review boards of the University of North Carolina (#16‐0276), University of Zambia (#001‐02‐16), James Cook University (#H6896) and University of South Carolina (#Pro00076701). All RCC participants provided written informed consent.

### 2.1 Study setting and population

To be eligible for the RCC study, participants had to be: incarcerated at one of five prisons in Lusaka or Central Province, Zambia; adults ≥18 years with documented HIV infection; scheduled for prison release within ∼30 days of study screening; enrolled in the national HIV treatment program [42, 43]; and, if on treatment, receiving antiretroviral therapy (ART) for ≥30 days. RCC participants were recruited, screened and enrolled prior to release, and underwent one baseline study visit prior to release and one follow‐up visit approximately 6 months post‐release. Baseline socio‐demographic and clinical information was collected at enrolment. The DCE survey was administered to consecutive RCC participants during the follow‐up visit. Our DCE sampling frame was limited to 125 participants who had not died, moved out of the study area, become lost to follow up or previously completed a follow‐up study visit during the DCE data collection period.

### 2.2 Discrete choice experiment

The DCE method is based on two theoretical foundations, Lancaster's theory [27] and RUT [25, 26]. Lancaster's theory postulates that a person chooses a good or service based on its intrinsic characteristics or attributes. The combination of attributes gives rise to utility or value. RUT assumes that utility is a latent, unobservable variable; an individual's choice of a good or service follows a stochastic process in which the utility of the chosen alternative matches or exceeds that of all alternate goods or services considered. A DCE simulates real‐world choice situations by asking participants to choose between systematically designed combinations of attributes. An analysis of participants’ choices provides estimates of the contribution of each attribute to utility (value). A positive contribution to utility is consistent with a preference for a specific attribute. The DCE presented here was used to elicit the relative preferences for modifiable attributes of transitional HIV care services for releasees living with HIV in Zambia.

### 2.3 Attributes and levels

**Figure 1:**
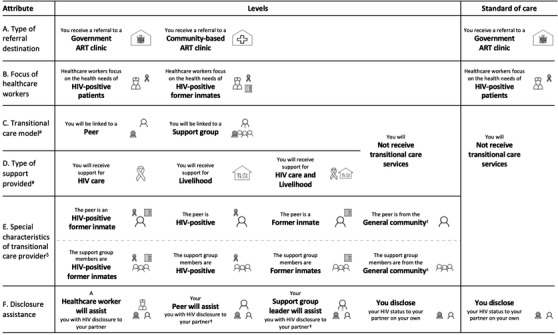
Characteristics of transitional HIV care evaluated in the DCE. Notes: # Non‐feasible level combinations of attributes C and D were excluded. The remaining combinations were included in the design as a seven‐level compound attribute. $ The visual and verbal description of attribute levels of E were dynamically matched to attribute C. ± This attribute level was considered as “no special characteristics” and paired with “no transitional care services”. † This attribute level was dynamically matched to attribute C. Abbreviations: ART, antiretroviral therapy; HIV, human immunodeficiency virus.

A list of preference‐relevant attributes of transitional HIV care services was generated based on the extant literature [19, 20, 22, 44, 45, 46, 47, 48, 49, 50, 51, 52, 53, 54, 55], preliminary results of a survey conducted with RCC participants about their transitional care experiences and results of five in‐depth interviews (IDIs) exploring RCC participants’ post‐release needs and experiences. A review of IDI results and expert judgement by the study team were used to prioritize six attributes for inclusion in the DCE. Each attribute was described by two to four feasible values commonly referred to as “levels” (Figure 1). Attribute levels were described verbally and using graphical depictions [56]. Pre‐tests with ∼50 individuals familiar with the transitional HIV care context for releasees in Zambia were used to iteratively refine the presentation of attributes and levels. Pre‐tests helped refine the descriptions of transitional care services (including translations) and led to the exclusion of unfeasible combinations of transitional HIV care characteristics. Unfeasible combinations included, for example, a pairing of not being linked to a transitional care model, but receiving transitional care services nonetheless (Appendix S1).

### 2.4 Experimental design and choice format

After excluding unfeasible combinations, the attribute levels depicted in Figure 1 yielded 296 feasible transitional care options, including the “standard of care” (SOC). The SOC, available to all releasees living with HIV in Zambia, was defined as: (1) referral to a government ART clinic with (2) healthcare workers who focus on the health needs of PLHIV generally, (3) no linkage to a transitional care provider and no receipt of transitional care services and (4) no assistance with HIV status disclosure to partners at release.

**Figure 2:**
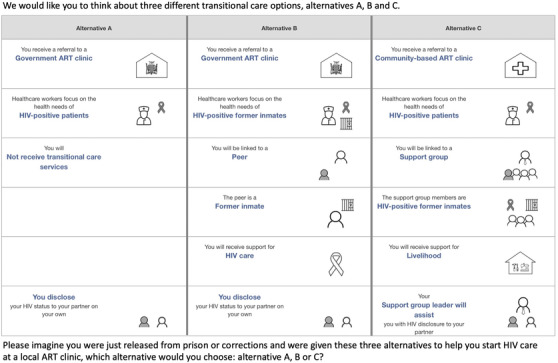
Sample choice task. Notes: after the most preferred option was selected, participants were asked to identify the next best option. The choice task was repeated with the most preferred option highlighted but not selectable. Participants were asked: “We would like you to think of the remaining two transitional care options. If today you were given these two remaining options in order to help you start HIV care at a local HIV clinic, which alternative would you choose?” The survey was translated into, and optimized for, the two most commonly spoken languages in Lusaka, Nyanja and Bemba.

The experimental design of a DCE represents the subset of choice tasks (selected from 43,660 potential choice tasks containing two feasible transitional care options and the SOC) that is used to estimate preference parameters with the smallest possible error [57]. Ngene software (ChoiceMetrics 2017) version 1.12b [58] was used to identify a D‐efficient design that was optimized for analysis using a mixed multinomial logit model with effects‐coded, normally distributed priors (Appendix S1). The direction and relative magnitude of priors were informed by IDIs and expert judgement by the study team. The final design included 84 choice tasks split into seven blocks with 12 tasks each. Participants were randomized across blocks, with the order of choice tasks randomized within participants and the order of alternatives randomized within tasks. Following a best‐best preference elicitation format [59], participants were asked in each task to identify their most preferred and next‐most preferred transitional care options. The DCE survey was translated into the two most commonly spoken languages in Lusaka, Nyanja and Bemba, and programmed into a custom‐built system for DCE administration on iOS tablet devices (Comet suite; Selway Labs 2019). A sample choice task is shown in Figure 2.

### 2.5 Survey administration

Trained local research assistants administered surveys in the participant's preferred language (English, Nyanja or Bemba) at a mutually agreed upon time and location. A paper survey was used to characterize participants’ post‐release experiences with healthcare and transitional care services (Data S1), while tablet devices were used to administer the DCE. First, participants were asked to read two short sentences to assess literacy [60], and visual acuity was assessed an E‐test [61]. Next, participants were provided with verbal and graphical descriptions of attributes and levels. For each attribute, participants were asked to rank the respective levels according to their preference. To ensure participant comprehension of DCE choice tasks, the results of these unconditional rankings of attribute levels were used to dynamically generate one training task with one dominant alternative (combining the participant’s preferred levels of all attributes) and one dominated alternative (combining less preferred levels of all attributes). Finally, each participant completed 12 DCE choice tasks. Additional questions assessed the difficulty of the survey and relative preferences for other support services not included in the DCE (for mental health, alcohol and drug use disorders).

### 2.6 Sample size

Our target sample size of *n* = 100 was based on a commonly used rule of thumb for estimating DCE sample size requirements [62, 63]. The formula n≥500c/ta combines the number of choice tasks *t*, alternatives per task *a* and the maximum number of levels per attribute *c* to derive a minimum sample size *n*. *A priori*, based on *t* = 12 choice tasks, *a* = 3 alternatives and *c* = 7 combinations of attributes C and D, we expected that ≥97.2 participants were required to characterize releasees’ average transitional care preferences.

### 2.7 Statistical analysis

Participants’ characteristics and unconditional rankings of attribute levels were analysed using descriptive statistics. DCE choice data, reflecting trade‐offs between different combinations of attribute levels, were analysed using a mixed logit model [64], with the choice of an alternative as the binary dependent variable and characteristics of the transitional care option (i.e. attribute levels) as independent variables whose coefficients were assumed to be normally distributed. Coefficient estimates from the mixed logit model represent estimates of participants’ average preferences for each transitional care characteristic relative to its reference level, which described the SOC. Coefficients’ estimated standard deviations describe the variation in preferences across participants [65, 66]. Standard deviations that are significantly different from zero indicate significant preference heterogeneity across releasees. Finally, individual participants’ choices were combined with information on the distribution of preferences across participants to derive individual‐level preference estimates (“posterior betas”) for each attribute level [64, 67, 68]. The range of the estimated individual‐level coefficients within attributes was compared across attributes to derive individual‐level estimates of relative attribute importance. A preliminary analysis of systematic preference heterogeneity involved the estimation of a series of mixed logit models in which the main effect for each attribute level was iteratively re‐estimated as fixed (instead of random), but with the model including an additional interaction between the attribute level and an observable characteristic. Statistical analyses were performed using STATA v16 (StataCorp, College Station, TX, USA).

## 3 RESULTS

In the parent RCC study, 296 participants were enrolled between 13 March 2017 and 31 December 2018. Of these, DCE surveys were conducted with 101 releasees (34.1%) participating in follow‐up visits from 9 January 2019—7 October 2019. Most DCE participants were male (*n* = 84, 83.2%) and had completed primary school (*n* = 54, 53.5%) (Table 1). Over one‐quarter of releasees (*n* = 29, 28.7%) were unemployed at follow‐up. Participants had spent an average of 8.2 months in the community prior to the DCE survey, with 18 (17.8%) reporting intervening re‐incarceration. Eighty‐seven participants (86.1%) reported being in HIV care at the time of DCE administration.

**Table 1:** Characteristics of participants at time of discrete choice experiment survey administration (*N* = 101)

		*n* (%)
Socio‐demographic characteristics		
Sex	Male (vs. female)	84 (83.2)
Age (years)	Median (IQR)	36.5 (32.3–43.8)
Marital status	Married/cohabitating/common law	63 (62.4)
	Divorced/separated	16 (15.8)
	Never married	16 (15.8)
	Widowed/other	6 (5.9)
Education (highest level completed)	Less than primary school	24 (23.8)
	Primary school completion	54 (53.5)
	Secondary schooling or higher	22 (21.8)
Employment since release	No work	29 (28.7)
	Formal employment	2 (2.0)
	Informal employment	42 (41.6)
	Self‐employed	28 (27.7)
Literacy^a^	Fully literate (vs. partially)	58 (57.4)
Survey language^a^	English	46 (45.6)
	Nyanja	47 (46.5)
	Bemba	8 (7.9)
Incarceration history		
Duration of incarceration at release (months)	Median (IQR)	12.0 (5.0–23.1)
Time since release (months)	Median (IQR)	8.2 (5.2–13.9)
Re‐incarceration after release	Yes (vs. no)	18 (17.8)
HIV history		
Months since HIV diagnosis	Median (IQR)	31.0 (18.3–81.6)
In HIV care	Yes (vs. no)	87 (86.1)
Total time on ART (months)	Median (IQR)	30.5 (18.2–79.3)
Self‐reported ART interruption since release	Yes (vs. no)	11 (10.9)

Abbreviations: ART, antiretroviral therapy; HIV, human immunodeficiency virus; IQR, interquartile range.

^a^ Assessed in the discrete choice experiment; all other characteristics were assessed as part of follow‐up procedures in the main study.

### 3.1 Direct assessment of transitional care preferences and receipt

**Figure 3:**
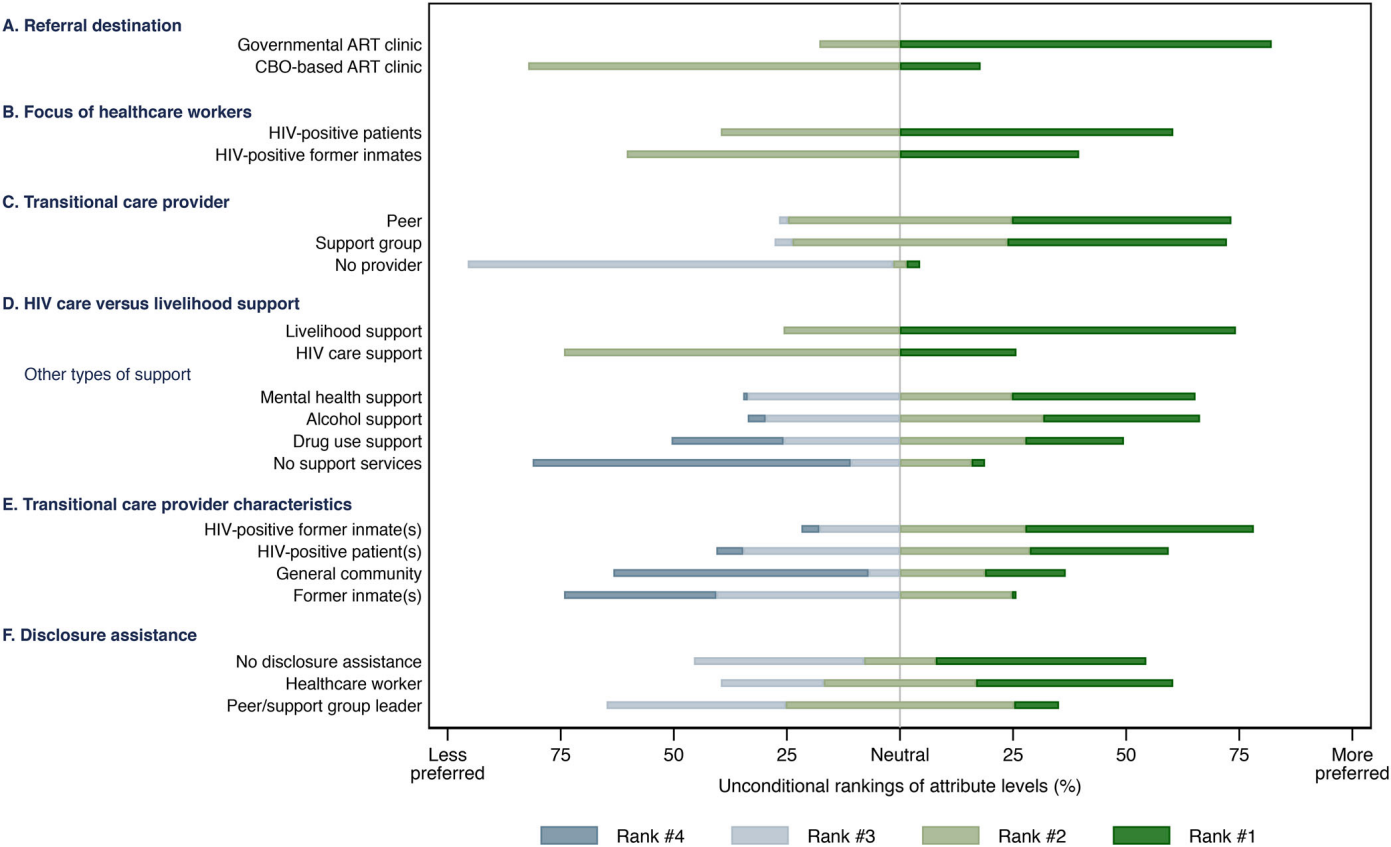
Rankings of transitional HIV care characteristics by recent releasees with HIV in Zambia (*N* = 101). Abbreviations: ART, antiretroviral therapy; CBO, community‐based organization; HIV, human immunodeficiency virus.

Figure 3 shows participants’ unconditional rankings of transitional care characteristics; Table 2 presents participants’ preferred transitional care characteristics against characteristics of care they actually received post‐release. Participants generally preferred referrals to government ART clinics (82.2%) and livelihood support (74.3%), and to be linked to either a support group (48.5%) or a peer navigator (48.5%), relative to no transitional care provider (3%). Substantial heterogeneity was observed in participants’ rankings of characteristics of the transitional care provider, the focus of healthcare workers and disclosure assistance options. More releasees expressed a preference to have formerly incarcerated PLHIV (50.5%) be their transitional care providers rather than PLHIV without an incarceration history (30.7%), formerly incarcerated persons without HIV infection (1%) or persons without either an incarceration or HIV history (17.8%). When asked about their preferences for services other than HIV care and livelihood support services, mental health services were the most commonly preferred (40.6%), followed by support for alcohol (34.6%) and drug use (21.8%) disorders. Nearly half of participants (46.5%) preferred no assistance with voluntary HIV status disclosure to their partners, while 43.6% preferred assistance from a healthcare worker.

**Table 2:** Characteristics of preferred and received transitional care services (*N* = 101)

			Preferred characteristics		Received characteristics		Concordance of preferred and received characteristics^a^	
			(*N* = 101)		(*N* = 101)			
			*n*	%	*n*	%	*n*	%
Linkage to local HIV care and treatment								
	Type of referral clinic							
		None	0	0.0	14	13.9	—	
		Governmental ART clinic	83	82.2	70	69.3	68	81.9
		Community‐based organization (CBO) ART clinic	18	17.8	17	16.8	14	77.8
	Client focus of healthcare workers							
		Focus on people living with HIV generally^b^	61	60.4	53	60.9	35	57.4
		Focus on releasees living with HIV specifically	40	39.6	1	1.2	0	0.0
		Don't know/refuse	—		33	37.9	—	
		N/A (not linked to care)	—		14	13.9	—	
Transitional care services								
	Transitional care model							
		Support group	49	48.5	1	1	1	2.0
		Peer navigator	49	48.5	12	11.9	11	22.4
		No provider	3	3	88	87.1	3	100.0
	Characteristics of transitional care providers							
		People living with HIV	31	30.7	12	11.9	8	25.8
		Formerly incarcerated person(s)	1	1	0	0.0	0	0.0
		Formerly incarcerated person(s) living with HIV	51	50.5	0	0.0	0	0.0
		Neither/general community member(s)^b^	18	17.8	1	1	0	0.0
		N/A (no provider)	—		88	87.1	—	
	Transitional care support services							
		Livelihood support	75	74.3	5^c^	5	3	4.0
		HIV care support	26	25.7	—		—	
	Other support services^d^							
		General mental health support	41	40.6	1^c^	1	0	0.0
		Support for hazardous alcohol use	35	34.6	2^c^	2	2	5.7
		Support for hazardous drug use	22	21.8	0	0	0	0.0
		No additional support services	3	3	14	13.9	0	0.0
Voluntary partner HIV status disclosure								
	Disclosure assistance type							
		No disclosure attempted	—		27	26.7	—	
		No assistance (i.e. self‐disclosure)	47	46.5	45	44.6	31	66.0
		Assisted by healthcare worker	44	43.6	28	27.7	23	52.3
		Assisted by peer navigator/support group leader	10	9.9	0	0	0	0.0
		Other assistance	—	—	1	1	—	

Abbreviations: ART, antiretroviral therapy; HIV, human immunodeficiency virus.

^a^ Concordance of preferred and received characteristics: number and proportion of people who received what they preferred; percentages related to the total number of people who preferred a characteristic.

^b^ Difference in the wording of response options for preferred and received characteristics.

^c^ Multiple responses possible.

^d^ Services not included in the discrete choice experiment.

—Indicates not applicable or not assessed.

Asked about their actual transitional care experiences post‐release, most participants (86.1%) reported having linked to a government ART clinic, primarily clinics that did not focus specifically on the healthcare needs of formerly incarcerated people (60.9%). Few participants reported receiving support from a peer navigator (11.9%) or support group (1%), and fewer still received livelihood support (5%) or support for hazardous alcohol use (2%) or other mental health issues (1%). Of 74 participants who had disclosed their HIV status to their partner(s), 45 did so without support from a healthcare worker.

### 3.2 DCE preference estimates

Table 3 shows results from the DCE choice data analysis. The estimated coefficients describe the average value (i.e. utility) derived from each transitional care characteristic in comparison with the SOC. Positive coefficients indicate a preference for the specific characteristic, while negative coefficients indicate an aversion, relative to the respective characteristic of the SOC. Eight of 11 transitional care characteristics were significantly preferred over the SOC. While healthcare workers’ focus on the health needs of releasees living with HIV (*p* = 0.583) and assistance with HIV serostatus disclosure from a peer navigator or support group leader (*p* = 0.939) were not significantly associated with preferences, linkage to care at a community‐based organization (CBO)‐run ART clinic was significantly less preferred (*p*<0.001), compared to the SOC. On average, transitional care providers living with HIV were most preferred, while the most preferred type of support, on average, was livelihood support provided through a support group. Across 1212 choice tasks, the SOC was selected as the most preferred option only 99 times (8%); it was ranked last 881 times (73%; not shown).

**Table 3:** Results of a mixed logit analysis of the discrete choice experiment data (*N* = 1212 best‐best choices by 101 participants)

			Average effect on preferences			Variation across participants	
			(“Mean preference”)			(“Preference heterogeneity”)	
Attribute	Level		Coefficient^a^	CI	*p*‐value	SD^b^	*p*‐value
A. Referral clinic type							
	Governmental ART clinic		ref.				
	Community‐based organization ART clinic		–0.78	[–1.00; –0.56]	<0.001	0.67	<0.001
B. Client focus of healthcare workers							
	Health needs of people living with HIV generally		ref.				
	Health needs of releasees living with HIV specifically		0.07	[–0.18; 0.32]	0.589	0.96	<0.001
C. Transitional care model and							
D. Type of support provided^c^							
	No transitional care support		ref.				
	HIV care support^d^	Peer navigator	0.68	[0.41; 0.94]	<0.001	0.81	<0.001
		Support group	1.00	[0.73; 1.28]	<0.001	0.63	<0.001
	Livelihood support^d^	Peer navigator	0.99	[0.72; 1.27]	<0.001	0.60	<0.001
		Support group	1.19	[0.92; 1.47]	<0.001	0.84	<0.001
E. Characteristics of the transitional care provider							
	Neither/general community member(s)		ref.				
	Formerly incarcerated person(s)		0.65	[0.40; 0.91]	<0.001	0.20	0.315
	Person(s) living with HIV		1.24	[0.99; 1.49]	<0.001	0.31	0.076
	Formerly incarcerated person(s) living with HIV		1.09	[0.82; 1.37]	<0.001	0.89	<0.001
F. Voluntary partner HIV disclosure assistance							
	No assistance (i.e. self‐disclosure)		ref.				
	Assisted by healthcare worker		0.27	[0.07; 0.48]	0.010	0.34	0.010
	Assisted by transitional care provider		–0.01	[–0.31; 0.28]	0.939	0.82	<0.001

Abbreviations: ART, antiretroviral therapy; CI, confidence interval; HIV, human immunodeficiency virus; ref, reference level (standard of care); SD, standard deviation.

^a^ Coefficients describe the “average utility” derived from each transitional care characteristic relative to the respective reference category, confidence intervals describe the precision with which the average is estimated.

^b^ Standard deviations describe the variability around the average utility derived from each transitional care characteristic across participants. The corresponding *p*‐values characterize the statistical significance of preference heterogeneity.

^c^ Attributes C (transitional care model) and D (type of support provided) were included in the experimental design and analysis as a compound attribute due to their inseparable nature (i.e. transitional care services can only be provided if PLHIV are linked to a transitional care provider).

^d^ The utility from both HIV care support and livelihood support was not significantly different from HIV care support only (*p* = 0.301 for peers; *p* = 0.062 for support groups), therefore, only the main effects for the two types of support were included in the final model.

**Figure 4:**
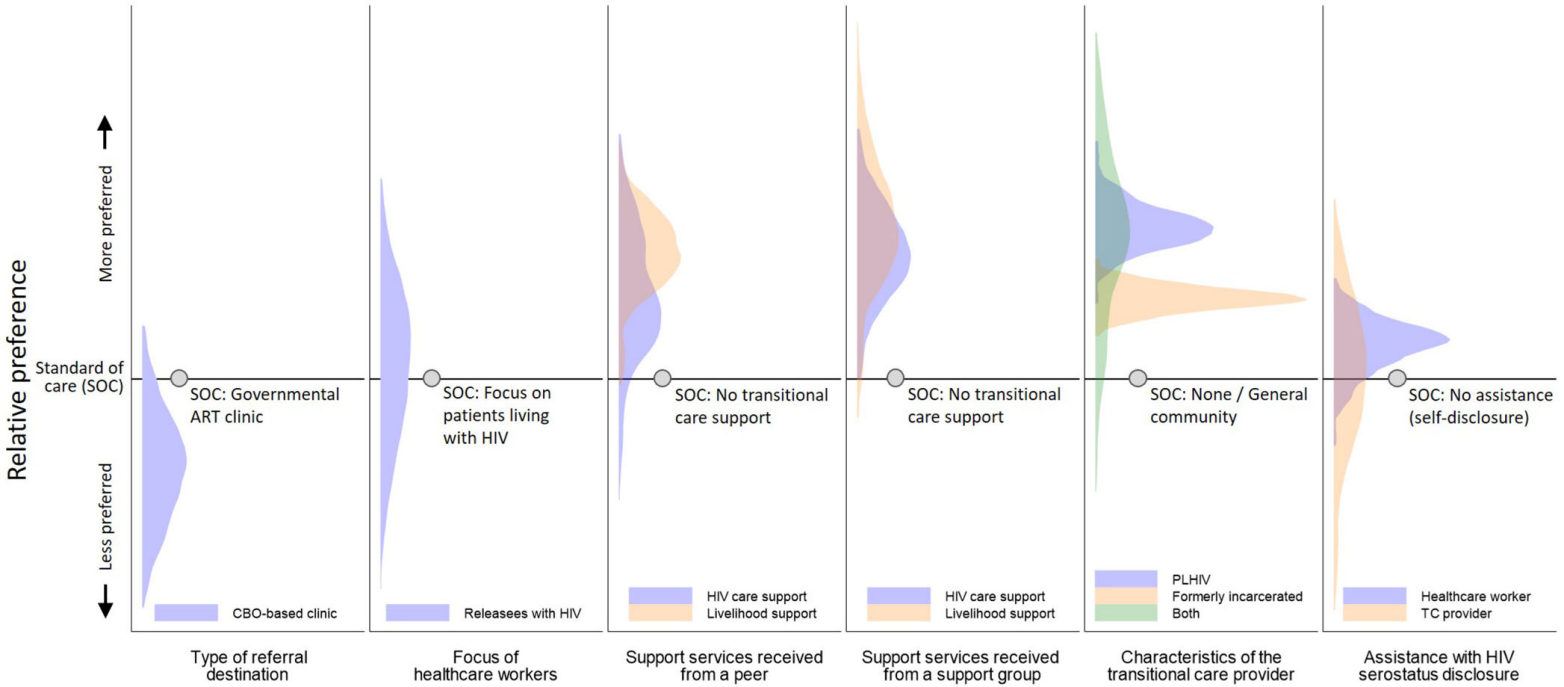
Distribution of individual‐level preferences for transitional care characteristics across participants (*N* = 101). Notes: shaded areas describe the distributions (kernel densities) of preference estimates for all 101 study participants. Values above the horizontal line indicate a preference for the respective transitional care characteristic relative to the standard of care; values below the line indicate a relative aversion. Shaded areas crossing the line indicate that some participants prefer the respective transitional care characteristic, while others prefer the standard of care. Preferences for attributes C (transitional care model) and D (type of support) are presented using separate panels for services received from a peer and a support group, respectively. Abbreviations: ART, antiretroviral therapy; CBO, community‐based organization; HIV, human immunodeficiency virus; PLHIV, people living with HIV; SOC, standard of care; TC, transitional care.

There was evidence of significant preference variation across participants, with the standard deviations for 9 of 11 attribute‐level coefficients significantly different from zero. For example, while the coefficient for healthcare workers’ focus on releasees living with HIV was not statistically significant, its standard deviation indicates significant variation in individual preferences within the sample, with some individuals perceiving healthcare workers’ focus on releasees living with HIV strongly negatively, while others viewing it strongly positively. Similar heterogeneity was observed for HIV status disclosure assistance from transitional care providers. The distributions of releasees’ preferences are shown graphically in Figure 4. Most releasees were averse to referrals to a CBO‐run ART clinic (vs. a government ART clinic), preferred livelihood support or HIV care support provided by a support group (vs. no support) and preferred transitional care providers who were either living with HIV or formerly incarcerated and living with HIV (vs. providers from the general community). Notably, negative correlations among individual‐level preference estimates for peers versus support groups (*ρ* = –0.07 and *ρ* = –0.09 for HIV care and livelihood support, respectively, results not shown) suggest differentiated preferences for these alternative transitional care models across participants. There was little evidence of systematic variation in preferences by participant characteristic, with only a handful of interaction terms found to be statistically significant (Appendix S1).

**Figure 5:**
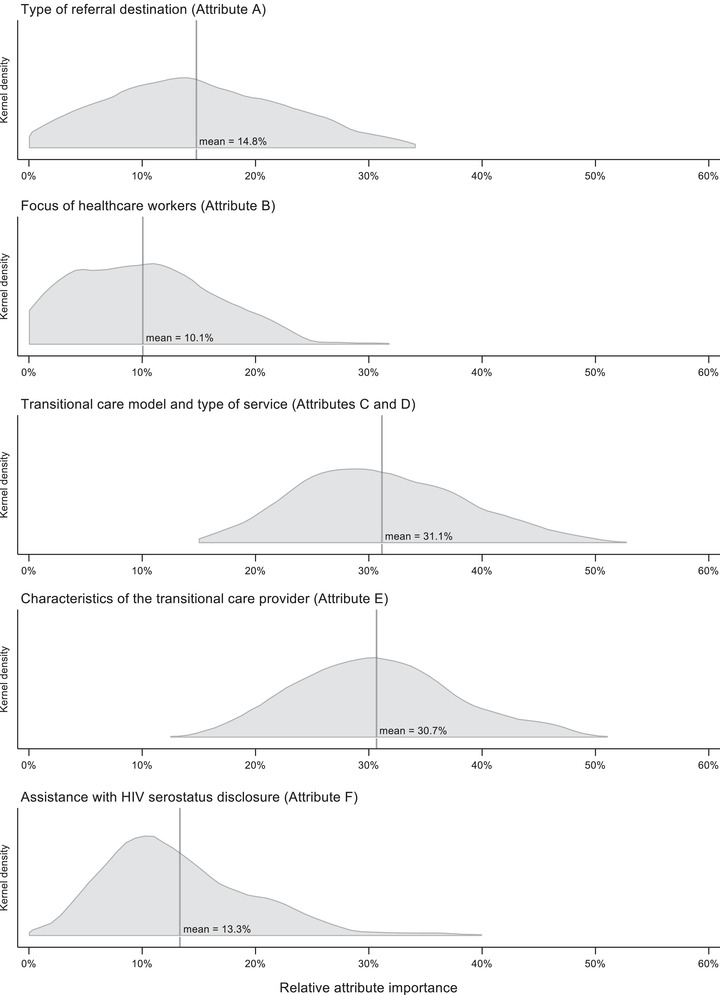
Distribution of relative attribute importance across participants (*N* = 101). Notes: individual‐level attribute importance was calculated using posterior preference estimates for each attribute level for each participant. The range of parameter estimates across the levels of an attribute reflects the attribute's potential effect on utility and was thus considered a measure of attribute importance. Relative attribute importance was calculated by dividing an attribute's range of parameter estimates (within the individual) by the sum of the ranges of parameter estimates across attributes, multiplied by 100. Owing to the inseparability of transitional care model and service type, the relative importance of these attributes is presented jointly. Abbreviation: HIV, human immunodeficiency virus.

Figure 5 summarizes the relative importance of transitional care attributes. The type and characteristics of the transitional care provider and the types of support received accounted jointly for nearly two thirds (62%) of the potential utility gains associated with different transitional care configurations. Referral destination, focus of healthcare workers and disclosure assistance, each accounted for 10–15% of potential utility gains. There was large variation in the relative importance of individual attributes across participants. Answers to the debriefing question assessing the difficulty of the DCE indicate that 4 participants (4%) found the DCE very difficult, 28 (27.7%) somewhat difficult and 69 (68.3%) not difficult (results not shown).

## 4 DISCUSSION

Findings from this DCE highlight the unmet need for transitional HIV care services in Zambia and opportunities for providing differentiated HIV care, livelihood and other support services to releasees living with HIV. Among the releasees we surveyed, virtually all transitional care characteristics evaluated were preferable to the SOC. A strong preference emerged for referrals to government ART clinics and services that help releasees access livelihood and HIV care support in their communities. The model of transitional care delivery also featured prominently, with support groups and peer navigators being preferred over current, non‐existent modalities. Experience living with HIV was the most preferred characteristic for transitional care providers. These results notwithstanding, we observed substantial variation in participants’ preferences, suggesting a role for differentiating services to meet the individual needs of releasees.

While, on average, livelihood assistance was the most preferred type of support, fitting with the needs of a study population in which many participants were unemployed, HIV care support for accompaniment to ART clinic visits and encouragement of medication adherence were also strongly preferred over the SOC. For both livelihood and HIV care support, a slight preference emerged for service delivery through a support group model, which is a common modality for providing psychosocial support to PLHIV in Zambia and SSA [69, 70]. Although support delivered by peer navigators was strongly favoured over no support, preferences were less strong relative to support groups. This may reflect the fact that peer navigators are not as commonly encountered in Zambia, or that peer providers are associated with back‐to‐care services typically reserved for patients who become lost to clinical follow up. Interestingly, participants who had not linked to HIV care in the community had a greater preference (compared to those who had linked) for livelihood support delivered by peer navigators, which may suggest a new role for peer navigators beyond their typical singular focus on HIV care support.

Participants stated a preference for continuing their HIV care in government ART clinics over clinics managed by CBOs catering to formerly incarcerated populations. This observation requires further study through our forthcoming qualitative analyses, and may reflect a number of explanatory factors, including: general community perceptions about the quality of health services in government ART clinics, a lack of ART services offered by most CBOs, a desire for anonymity and avoidance of stigma associated with services for formerly incarcerated people or the convenience and familiarity of collecting ART through government health facilities [41]. Interestingly, while releasees preferred referrals to government ART clinics for continuing HIV care once in the community, a substantial number of participants expressed a preference for receiving care from healthcare workers conversant with the unique needs of releasees living with HIV. Such a result may reflect a preference for client‐centred care more generally, or a specific desire for services responsive to the unique needs of releasees, particularly in the face of dual stigma posed by their HIV status and incarceration history [71]. While assistance with partner disclosure did not feature strongly in participant preferences, helping releasees voluntarily disclose their HIV status may help overcome barriers to family and community re‐integration. Through the scale up of community index testing, voluntary assisted partner notification services are increasingly becoming a major part of HIV testing services in Zambia [72], and may offer a natural platform for family counselling and partner HIV status disclosure for releasees.

Examining participants’ mean preferences and preference heterogeneity simultaneously reveals substantial individual variation in what releasees want from transitional care services, and argues against a “one size fits all” approach to transitional HIV care. That said, several overarching observations emerged that can inform the design of a future transitional HIV care intervention. First, there were clear releasee preferences for receiving HIV care and treatment at government ART clinics, which can serve as the foundation for transitional care programming and help marshal existing government human and clinical resources to enhance the health of releasees. Second, transitional care models involving support groups or peer navigators were generally strongly preferred over the SOC. With peer navigation emerging as a strategy to support HIV care continuity for key populations [73, 74], including for incarcerated people and releasees living with HIV [75], a hybrid approach involving options for both support groups and peer navigators may provide a differentiated means of meeting the heterogeneous preferences of this population. Third, the strong preference for livelihood support speaks to the need for transitional care interventions that go beyond HIV care support alone and that more holistically address basic needs like food, housing and employment. Finally, releasees expressed a preference for mental health services, including for mitigating hazardous alcohol and drug use, which are scarce in the current Zambian HIV treatment program.

We acknowledge several limitations. First, the use of hypothetical transitional care characteristics may have introduced measurement error, potentially biasing estimates of releasee preferences. Second, stated preferences may have varied from revealed preferences (i.e. participants’ actual decisions during and after release) due to multi‐level factors and constraints [76], such as health system barriers to community HIV care, family and partner relationship dynamics, and the demands of meeting basic needs. We attempted to mitigate these biases by concurrently administering a transitional care questionnaire grounded in participants’ actual post‐release experiences and by having research staff familiar with the study population administer the DCE. Third, given the virtually non‐existent literature on the post‐release experience of incarcerated PLHIV in SSA, there was limited evidence from the region to guide the selection of attributes, which may have led to the omission of some important attributes. However, the attributes selected align with the major themes identified through our formative IDIs. Fourth, given the considerable resources required for following participants after release, and high rates of recidivism in Zambia [11], we were unable to enrol a larger DCE sample. This may have resulted in selection bias, and limited the generalizability and precision of our estimates. Finally, most of our sample was in HIV care at the time of the DCE, and, as such, our results may not fully capture the preferences of releasees experiencing prolonged care disengagement.

## 5 CONCLUSIONS

Improving HIV programming for key populations, including formerly incarcerated PLHIV, requires partnering with these populations and rigorously documenting their HIV care needs and preferences. In the first DCE with releasees living with HIV in SSA, we identified preferred characteristics of transitional HIV care that can form the basis for differentiated service delivery models for this population. Such models should aim to provide longitudinal and individualized HIV care support to releasees, offer client‐centred treatment in trusted health facilities and strengthen linkages to programs and organizations providing livelihood and mental health support.

## COMPETING INTERESTS

The authors have no competing interests to declare.

## AUTHORS’ CONTRIBUTIONS

JO, VY and MEH had overall responsibility for implementing the study, conceived and designed the study, developed the study protocol, collected and analysed the data, and wrote the manuscript. HJS and CM contributed to developing the concept, design and protocol for the study. VY, HJS, MN, PC and LK contributed to data collection. VM assisted with data analysis and data interpretation. All authors reviewed the manuscript critically for intellectual content, and read and approved the final draft of the submitted manuscript.

## FUNDING

Research reported in this publication was supported by the Fogarty International Center of the National Institutes of Health under Award Number K01TW010272 (MEH). The content is solely the responsibility of the authors and does not necessarily represent the official views of the National Institutes of Health.

## Supporting information


**Appendix S1**: Discrete Choice Experiment (DCE) Appendices

**Data S1**: Transitional Care Preferences Questionnaire
