# Supplementary material for: Preferences for transitional HIV care among people living with HIV recently released from prison in Zambia: a discrete choice experiment
Source: J Int AIDS Soc. 2021 Oct 14;24(10):e25805. doi: 10.1002/jia2.25805 (PMC8516367; doi:10.1002/jia2.25805)
Supplement: Supplementary file 1 — Appendix S1: Discrete Choice Experiment (DCE) Appendices [file JIA2-24-e25805-s002.docx]

**Discrete Choice Experiment (DCE) Appendices**

[Appendix 1: List of excluded combinations of attribute levels 2](#_Toc75772398)

[Appendix 2: Design and analysis of the DCE 3](#_Toc75772399)

[1 Decision model 3](#_Toc75772400)

[1.1 Attributes 3](#_Toc75772401)

[1.2 Levels 3](#_Toc75772402)

[1.3 Standard of care alternative 4](#_Toc75772403)

[1.4 Presentation of attributes and levels 4](#_Toc75772404)

[2 Preference Elicitation Technique 6](#_Toc75772405)

[2.1 Choice context 6](#_Toc75772406)

[2.1.1 Primary choice context 6](#_Toc75772407)

[2.1.2 Secondary choice context 6](#_Toc75772408)

[2.2 Choice tasks 6](#_Toc75772409)

[3 Experimental Design 7](#_Toc75772410)

[3.1 Identification of an efficient design 7](#_Toc75772411)

[3.2 Evaluation of the design 8](#_Toc75772412)

[3.3 Assignment of choice tasks to subjects 8](#_Toc75772413)

[3.4 Sample size 8](#_Toc75772414)

[4 Instrument Design 9](#_Toc75772415)

[4.1 DCE survey design 9](#_Toc75772416)

[4.2 Pre-testing 9](#_Toc75772419)

[5 Data Collection 10](#_Toc75772420)

[5.1 Recruitment strategy 10](#_Toc75772421)

[5.2 Mode of survey administration 10](#_Toc75772422)

[5.3 Protocol registration 10](#_Toc75772423)

[6 Data Analysis 11](#_Toc75772424)

[6.1 Statistical model 11](#_Toc75772425)

[6.2 Interpretation and presentation of results 11](#_Toc75772426)

[7 References 12](#_Toc75772427)

# Appendix 1: List of excluded combinations of attribute levels

| Attributes | TC provider | Type of support | Characteristics of TC provider | Disclosure assistance |
| --- | --- | --- | --- | --- |
| Unfeasible combinations  of attribute levels | No provider | HIV care support |  |  |
|  | No provider | Livelihood support |  |  |
|  | No provider | HIV & livelihood support |  |  |
|  | No provider |  | Former incarcerated person(s) living with HIV |  |
|  | No provider |  | People living with HIV |  |
|  | No provider |  | Former incarcerated person(s) |  |
|  | No provider |  |  | TC provider |

TC = transitional care; HIV = Human immunodeficiency virus

# Appendix 2: Design and analysis of the DCE

# Decision model

## Attributes

Extant literature, preliminary results from a transitional care survey and in-depth interviews, as well as expert judgment by members of the study team were used to select six characteristics of transitional care (TC) services for inclusion in the decision model:

- Type of referral destination
- Focus of healthcare workers
- Disclosure assistance
- TC provider
- Characteristics of TC provider
- Type of support

In the experimental design and statistical analysis, these six characteristics were operationalized using five attributes:

- Type of referral destination
- Focus of healthcare workers
- Disclosure assistance
- TC provider and type of support *(combining transitional care provider and type of support)*
- Characteristics of TC provider

## Levels

**Type of referral destination.** The type of referral destination was described by a 2-level attribute:

1. You receive a referral to a government ART clinic
2. You receive a referral to a community-based ART clinic

**Focus of healthcare workers.** Focus of healthcare workers was described by a 2-level attribute:

1. Healthcare workers focus on the health needs of HIV-positive patients
2. Healthcare workers focus on the health needs of HIV-positive former inmates

**Disclosure assistance.** Disclosure assistance was described by a 3-level attribute (Display of *peer* or *support group leader* were based on the level specification of **TC provider**):

1. A healthcare worker will assist you with HIV disclosure to your partner
2. Your *peer/support group* *leader* will assist you with HIV disclosure to your partner
3. You disclose your HIV status to your partner on your own

**TC provider.** The TC provider was described by a 3-level attribute:

1. You will be linked to a peer
2. You will be linked to a support group
3. You will not receive transitional care services (combined display of dependent attributes: TC provider, type of support, and characteristics of TC provider 🡪 later referred to as [Blank])

**Type of support.** Type of support was described by a 4-level attribute:

1. You will receive support for HIV care and livelihood
2. You will receive support for livelihood
3. You will receive support for HIV care
4. [Blank]

**Characteristics of TC provider.** Characteristics of the TC provider were described by a 4-level attribute (Display of *peer* or *support group members* and grammatical adjustments were based on the level specification of **TC provider**):

1. The *peer/support group members* is an/are HIV-positive former inmate(s)
2. The *peer/support group members* is/are HIV-positive
3. The *peer/support group members* is a/are former inmate(s)
4. The *peer/support group members* is/are from the general community **OR**

[Blank]

- - Note: This level represents the absence of any special characteristics of the TC provider and is therefore used as the respective level, when the participant will not receive transitional care services

## Standard of care alternative

The standard of care alternative, describing the current default transitional care option available to releasees consists of the following attributes and levels:

- **Type of referral destination:** You receive a referral to a government ART clinic
- **Focus of healthcare workers:** Healthcare workers focus on the health needs of HIV-positive patients
- **Disclosure assistance:** You disclose your HIV status to your partner on your own
- **TC provider/Type of support/Characteristics of TC provider:** You will not receive transitional care services

## Presentation of attributes and levels

Attributes were introduced one-by-one, with verbal and graphical descriptions of key attribute levels:

**Type of referral destination.** After your release from prison, you are encouraged to continue HIV care and treatment in the community. There are different types of clinics offering HIV care: government ART clinics and community-based ART clinics run by organizations in the community (such as churches, community-based organizations, and the like. [Followed by a ranking exercise]

**Focus of healthcare workers.** The clinics and their healthcare workers offer different services to their patients. Some clinics and their healthcare workers focus on the health needs of HIV-positive patients, others focus on the health needs of HIV-positive former inmates. [Followed by a ranking exercise]

**Disclosure assistance.** It is important that you tell your sexual partner or partners that you have HIV and encourage them to get tested for HIV. However, telling someone that you have HIV can be very difficult. We want you to consider three options for helping you tell your partner or partners that you have HIV. [Followed by a ranking exercise]

**TC provider.** The change from getting HIV treatment in prison or correction to getting HIV treatment in the community can be difficult. To help you continue with your HIV treatment after release, there are two types of people who can help you connect to HIV treatment in the community. You may be linked to a peer who is someone who successfully dealt with situations similar to yours in the past and who could be a mentor or friend to you. You may also be linked to a support group, which is a group of people sharing common experiences. Support group members share their problems and concerns and provide each other with encouragement, comfort, and advice. Alternatively, some people prefer to connect to HIV treatment and care on their own, without the help of a peer or support group. [Followed by a ranking exercise]

**Characteristics of TC provider.** (Display of description of TC characteristics for *peer* or *support group members* was based on the ranking response to **TC provider**; every participant was presented with the characteristics of their individually preferred TC provider)

A peer usually has things in common with you. She or he may share the experience of being from your community, living with HIV, having been previously incarcerated, or all of these experiences. We want you to think of 4 different types of peers. [Followed by a ranking exercise]

**OR:** Support group members usually have things in common. They can share the experience of being from your community, living with HIV, having been incarcerated, or all of these experiences. We want you to think of 4 different types of support groups. [Followed by a ranking exercise]

**Type of support.** Peers and support groups offer several types of services. We want you to think of 2 different services offered by peers and support groups. HIV care support includes sharing experiences on living with HIV and taking ARVs, adherence counseling, and getting information about positive living and how to stay healthy. It can also include help on finding an ART clinic and having someone accompany you to your first few visits. Livelihood support includes services that help with getting you back on your feet in the community and taking care of basic necessities like help finding housing and work. [Followed by a ranking exercise]

# Preference Elicitation Technique

## Choice context

In each choice task, participants were asked to identify their most- and next-most preferred transitional care options (“best-best” elicitation method).^1^

### Primary choice context

In each choice tasks, participants were given the following instructions:

*We would like you to think about three different transitional care options, alternative A, B and C.* [Presentation of 3 unlabeled transitional care options]. *Please imagine you were just released from prison or correction and were given these three alternatives to help you start HIV care at a local ART clinic, which alternative would you choose: alternative A, B or C?*

### Secondary choice context

After selecting the most preferred transitional care option, the remaining options were presented again. Participants were given the following instructions:

*We would like you to think of the remaining two transitional care options, that you did not choose in the first place.* [Presentation of 3 unlabeled transitional care options; the previously chosen, most preferred transitional care option was indicated with a green background; participants were unable to re-choose the previously chosen option]*. If today you were given these two remaining options in order to help you start HIV care at a local HIV clinic, which alternative would you choose?*

## Choice tasks

Each respondent’s rankings of levels for all attributes were used to populate a respondent-specific comprehension task with clearly dominant (preferred levels for all attributes), dominated (worse levels for all attributes) alternatives.

The comprehension task was followed by 12 DCE questions, or choice tasks. In each task, participants were shown two transitional care options and the standard of care option. Participants were asked to select their preferred and next-preferred transitional care options.

Choice tasks represented unforced-choice scenarios. Alternatives were unlabeled. All attributes were included in each choice task. A sample choice task is shown in **Figure 2**.

# Experimental Design

## Identification of an efficient design

The combination of transitional care characteristics across the 6 attributes (2 type of referral destination options x 2 focus of healthcare workers options x 3 disclosure assistance options x 7 combinations of transitional care provider and types of support x 4 characteristics of TC provider options) yielded 336 potential transitional care options. Out of these 336 options, 40 infeasible options were excluded to rule out implausible combinations (see Appendix 1). The remaining 296 transitional care options in turn, resulted in 43660 potential choice tasks comprised of two distinct transitional care options and the standard of care.

Ngene software (ChoiceMetrics 2017) version 1.12b was used to select from these 43660 potential choice tasks a *d*-efficient design (MNL D-error 0.095695; Panel MNL D-error=1.212144) consisting of 84 tasks; the design was optimized for analysis using a mixed multinomial logit model with effects-coded, normally distributed priors; 100 Halton draws were used to evaluate candidate designs over the parameter distributions. Priors (mean, standard deviation) were based on preliminary results from a transitional care survey and in-depth interviews, as well as expert judgment by members of the study team.

Type of referral destination: Governmental clinic (0, 1)

Community-based clinic (reference level: 0, 1)

Focus of healthcare workers: Focus on HIV-positive patients (0.5, 1)

Focus on HIV-positive former inmates (reference level: -0.5, 1)

Disclosure assistance: Healthcare worker (0.5,1)

Peer/Support group leader (0, 1)

No assistance (reference level: -0.5,1)

TC provider and type of support: Peer & both support services (0.1, 1)

Peer & HIV care support (0.1, 1)

Peer & livelihood support (0.1, 1)

Support group & both support services (0.1, 1)

Support group & HIV care support (0.1, 1)

Support group & livelihood support (0.1, 1)

No provider & no support services (reference level: -0.6, 1)

Characteristics of TC provider: HIV-positive former inmate(s) (0.5,1)

HIV-positive (0, 1)

Former inmate(s) (0, 1)

General community (reference level: -0.5,1)

## Evaluation of the design

The design did not include dominant alternatives (the highest estimated choice probability for any alternative across the 84 choice tasks was 0.779) and low correlations were observed between attribute levels (the highest correlation within non-standard of care alternatives was <0.30, the highest correlation between non-standard of care alternatives was <0.50).

**Table A1. Correlations between attribute levels within and across alternatives**

## Assignment of choice tasks to subjects

Participants were randomized across 7 blocks with 12 tasks each; the order of choice tasks was randomized across participants, and the order of transitional care options was randomized within each task.

## Sample size

The target sample size of n=100 for this study was calculated using a commonly used rule of thumb for the minimum sample size for a DCE:^2^

$\frac{n t a}{c}$ ≥ 500

with *n* representing the sample size, *t* representing the number of choice tasks per participant, *a* representing the number of alternatives in each choice task, and c representing the highest number of attribute level of any attribute included in the decision model.

In this study (with t = 12, a = 3, and c = 7), a minimum sample size of 97.2 participants was suggested using Orme’s formula.

An empirical power-test formula by Yang et al.^3^, applied to the DCE design employed in this study, suggests that a sample of N=100 allowed us to estimate the utility difference between the most and least-preferred profiles with a standard deviation of ~0.4, which appears to be roughly in line with an ‘average’ DCE study. The very efficient design (d-error 0.09), above average number of choice tasks (12), lack of probabilistic attributes, and the inclusion of a 3rd alternative for a best-best choice scenario appear to partially compensate for the comparatively low sample size.

# Instrument Design

## DCE survey design

DCE surveys were designed for administration on iPad Pro devices using the custom-built *comet* suite (Selway Labs 2019), comprised of *comet-control*, a web application for managing survey content, *comet-engine*, a JavaScript application for displaying surveys and collecting data, and *comet-client-ios*, a native iOS app for downloading updates to comet-engine and survey content and for uploading completed surveys.

The survey content, including attribute introductions, verbal and graphical descriptions of attribute levels, and choice scenarios implied by the DCE’s experimental design, were coded as JSON objects and imported into the *comet-control* web application. The DCE survey was translated into the two most commonly languages spoken in the study area, Nyanja and Bemba. The *comet-engine* interpreted these objects and rendered the DCE survey on 12.9-inch iPad Pro devices.

## Pre-testing

The DCE survey was pre-tested with approximately 50 participants, resulting in changes to the wording, translations, and graphical display of attribute levels to aid participant comprehension. Participants involved in pre-testing were not included in the DCE survey.

# Data Collection

## Recruitment strategy

All DCE participants are participants of the larger parent study: Releasee Care Continuum (RCC) study. To be eligible for the parent RCC study, potential participants had to be currently incarcerated at 5 prisons located in Lusaka and Central Province, Zambia, adults ≥18 years of age, with documented HIV infection, enrolled in the national HIV program, and scheduled for prison release within approximately 30 days; eligible incarcerated persons on antiretroviral therapy (ART) had to be nominally on treatment for ≥30 days. RCC participants were recruited, screened, and enrolled prior to release, and underwent one baseline pre-release and one follow-up post-release study visit (scheduled for approximately 6 months post-release). The DCE survey was administered to consecutive RCC participants during the follow-up visit.

## Mode of survey administration

Surveys were administered using iPad devices, in-person, by trained research staff.

## Protocol registration

The DCE is part of the Releasee Care Continuum (RCC) study. RCC activities, including the DCE, were approved by the ethics committees of the University of North Carolina, USA (#16-0276), the University of Zambia (#001-02-16), and James Cook University, Australia (#H6896). Analysis of de-identified DCE preference data was exempt by the Health Sciences South Carolina Institutional Review Board (#Pro00076701).

# Data Analysis

## Statistical model

The DCE data are comprised of 12 stated choices per participant, each indicating a preference among 2 varying transitional care options and the standard of care. Analysis and interpretation of the DCE data are based on a random utility model, with the utility individual *i* derives from alternative *a* described by $U_{ia}=V\left( X_{ia}\beta\right)+\varepsilon_{ia}$. *X* describes a vector of transitional care characteristics, while *β* is a vector of taste parameters. The specifications of $V\left( X_{ia}\beta\right)$ and $\varepsilon_{ia}$ vary based on the specific estimation model.

To estimate mean (average) preferences and standard deviations expressing the magnitude of preference variation in the studied sample, DCE data were analyzed using mixed, or random parameters, logit models in Stata version 16 (StataCorp, College Station, Texas).^4^ The significance of two-way interactions between attributes was analyzed and resulted in a model that included main effects for 4 attributes (type of referral destination, focus of health care workers, characteristics of the transitional care provider, and disclosure assistance) and interactions of the two remaining attributes (transitional care model and type of support provided). The parameter estimates on the three-way interactions between (a) both HIV care and livelihood support and (b) transitional care model (peer vs. support group) were not significantly different from those of HIV care only and were thus not included in the final model.

## Interpretation and presentation of results

Average preferences for each attribute level are described by dummy-coded parameter estimates (i.e., coefficients) from a mixed logit model (Table 3). Standard errors describe the precision with which average preferences are estimated. Standard deviations of the mean preferences for each attribute level describe the variation across participants. Information on the distribution of preferences across participants was subsequently combined with information on individual participants’ choices to derive individual-level (“posterior”) preference estimates for each attribute level.^4-6^ The distributions of preference estimates for each attribute level, relative to the respective standard of care levels, are presented graphically (**Figure 4**).

The maximum range of utilities across all the levels of an attribute, i.e., the difference between the most and least preferred attribute levels, can be interpreted as a measure of an attribute’s importance. Across attributes, relative attribute importance was therefore calculated as the share of the sum of absolute attribute importance weights attributable to each attribute (expressed as a percentage). Because estimated preference weights (and thus the ranges across levels within each attribute) varied across participants, the distribution of relative attribute importance similarly varies across participants. These distributions are shown in **Figure 5**.

## Analysis of systematic variation in preferences

A preliminary analysis of systematic preference heterogeneity involved the estimation of a series of 44 mixed logit models in which the main effect for each of the 11 attribute levels was iteratively re-estimated as fixed (instead of random) but with the model including an additional interaction between the attribute level and an individual characteristic. The participant characteristics of interest were:

- female gender (vs. male),
- younger age (<35 years vs. 35 years or older),
- no or unknown linkage to HIV care since release (vs. linkage to care), and
- no or unknown disclosure of the HIV status to the partner (vs. self-reported disclosure).

These variables varied meaningfully across participants and are plausibly associated with transitional care preferences. Statistical significance of the coefficient estimate for the interaction term is indicative of systematic variation in preferences for the respective attribute level with the respective characteristic of the participant.

Results suggest limited systematic variation in preferences with the participant characteristic evaluated. Only 4 of the 44 interaction terms were statistically significant at conventional levels (Table A2).

**Table A2. Systematic variation in preferences with participant characteristics**

Notes: See methods above.

Coefficients and 95% confidence intervals on interaction terms from 44 separate mixed logit models. * and ** indicate statistical significance at the 0.05 and 0.01 levels, respectively.

ref – reference level; CI – confidence interval; ART – antiretroviral therapy

# References

1. Ghijben P, Lancsar E, Zavarsek S. Preferences for oral anticoagulants in atrial fibrillation: a best-best discrete choice experiment. *Pharmacoeconomics.* 2014;32(11):1115-1127.

2. Orme B. Sample size issues for conjoint analysis. In: Orme B, ed. *Getting Started with Conjoint Analysis. Strategies for Product Design and Pricing Research* Second ed. Madison, WI: Research Publishers LLC; 2010:57-65.

3. Yang JC, Johnson FR, Kilambi V, Mohamed AF. Sample size and utility-difference precision in discrete-choice experiments: A meta-simulation approach. *J Choice Model.* 2015;16:50-57.

4. Hole AR. Fitting mixed logit models by using maximum simulated likelihood. *The Stata Journal.* 2007;7(3):388-401.

5. Revelt D, Train K. Customer-specific taste parameters and mixed logit: Households' choice of electricity supplier. 2000.

6. Train KE. Individual-Level Parameters. In: *Discrete choice methods with simulation.* Cambridge, MA: Cambridge university press; 2003:262-283.
