# Supplementary material for: Preferences for transitional HIV care among people living with HIV recently released from prison in Zambia: a discrete choice experiment
Source: J Int AIDS Soc. 2021 Oct 14;24(10):e25805. doi: 10.1002/jia2.25805 (PMC8516367; doi:10.1002/jia2.25805)
Supplement: Supplementary file 2 — Data S1: Transitional Care Preferences Questionnaire [file JIA2-24-e25805-s001.pdf]

UIC: □□-□□-□□-□□

First 2 letters of First Name-2 Digit birth year-First 2 letters of Surname-Birth order

Transitional Care Preferences Questionnaire

Understanding Longitudinal Clinical Outcomes and Post-release Retention in Care among HIV-infected Inmates in Lusaka, Zambia

Continuum Study

PLEASE NOTE FOLLOWING INCLUSION AND EXCLUSION CRITERIA

Inclusion criteria:

- Inmates at Lusaka Central Prison
- Adult prisoners ≥18 years of age
- Documented HIV infection
- Release scheduled or otherwise anticipated within 30 days of study enrollment
- Enrolled in the national HIV programme while incarcerated (i.e. receiving ART, pre-ART care, or otherwise followed by the national HIV programme)
- If on treatment, has been receiving ART for ≥3 months
- Plan to live in Lusaka Urban District after release
- Willing and able to provide locator information, including a reachable telephone number
- Willing and able to provide informed consent in a study language (Bemba, Nyanja or English)

Exclusion criteria

- Unavailable at the time of the questionnaire
- Residing outside Lusaka district before incarceration
- Remandees awaiting trial who have been detained less than 180 days
- Offenders being held at Lusaka Central Prison while awaiting appeal of a capital offence (e.g. murder or rape) or who have had their current sentence extended for violent behavior during their period of incarceration
- Not able to provide informed consent

UIC: □□-□□-□□-□□

First 2 letters of First Name-2 Digit birth year-First 2 letters of Surname-Birth order

Continuum Transitional Care Questionnaire – 1.2

1. Questionnaire Administration Date…………………………………	□□/□□□/□□□□<br>dd / MMM / yyyy
2. Interviewer ID…………………………………………………………………………<br>[intid]	□□□□<br>***(**required field**)***
3. Interview language……………………………………………………………………<br>[lang] 0=English 1=Nyanja 2=Bemba	□

Introduction:

[Read Verbatim]: "Our Continuum study team is interested in knowing about the health of people living with HIV both while they are in prison/ corrections and after they leave prison/ corrections. We are interested to hear your opinions, thoughts and preferences about how best to support people living with HIV as they leave prison / releasees. We want to know about what types of health and support services you would have liked to have received just before release, upon re-entry in the community, and in the weeks and months thereafter.

You do not have to answer any question that you do not want to. Some of these questions are sensitive, but we ask them because it is important that we understand your feelings, experiences, and preferences so that we can better understand how best to support people once they leave prison/ corrections and return to the community. All of your answers will be kept confidential. Confidential means we will not tell your answers to anyone outside the research group and we will not share any of your answers with correctional officers or parole board officials.

Please be honest when answering these questions and take your time when answering. There are no right or wrong answers. If you are not sure what a question means, then I will be happy to explain. Again, you don't have to answer any question if you don't want to and you may stop the questionnaire at any time. If any of the questions make you feel uncomfortable, please just say 'I don't want to answer that at the moment.'

Do you have any questions so far?"

SECTION I: DISCHARGE SERVICES
(8 items, 12 questions)

**Domains**: *Availability; Releasee experiences with re-integration into the community and mainstream HIV services after prison discharge*	1. ***[Read Verbatim]:*** In this section, I will be asking you questions about your most recent experience being discharged from the correctional facility/ prison. To start, I will read you a list of services and other offerings that are currently provided to inmates before discharge / release. Looking back based on your experience trying to get HIV care and treatment in the community after your most release/ discharge, which ones did you find most helpful/ useful?<br><br>***[READ responses aloud and Mark <u>ALL</u> that apply]:***<br>☐ 0) Having a referral letter addressed to the health facility<br>☐ 1) Receiving a Smart Card / plastic card with their medical history<br>☐ 2) Pre-release Counselling from the Corrections Officer / Health Worker<br>☐ 3) Contact details for a Parole Board Member<br>☐ 4) 30- or 60-day supply of medicines (ARVs, CPT, TB medications)<br>☐ 5) Referral to an organization helping inmates (e.g. Chreso, PRISCCA, etc.)<br>☐ 6) Anything else not listed here that was helpful when you tried to access HIV care and treatment in the community? Please specify: ____<br>☐ 7) I have not tried to access HIV care and treatment in the community yet.
**Domains:** *Releasee experiences with re-integration into the community and mainstream HIV services after prison*	2. ***[Read verbatim, filling in the names of the services/ offerings provided under question #1 above]:*** Please give at least one reason why you found ***<u>[fill in 1st option from question #1 above]</u>*** helpful?<br><br>***[Use free listing method every time a participant gives a new reason, as follows]:***<br>You mentioned ***<u>[fill in reason given]</u>*** as a reason you found ***<u>[fill in first option from question #1 above]</u>*** helpful, was there any other reason you found ***<u>[fill in same option]</u>*** helpful?<br><br>***[Continue summarizing the reasons given by the participant for the first option from question #1 above until the participant provides no more new reasons. Then proceed to the next option from question #1***

above, and repeat the process].

Please give at least one reason why you found ***[fill in 2nd option from question #1 above]*** helpful?....

Reasons for First service/ offering from question #1 above

i)……

ii)……

iii)……

Reasons for Second service/ offering from question #1 above

i)……

ii)……

iii)……

Reasons for Third service/ offering from question #1 above

i)……

ii)……

iii)……

Reasons for Fourth service/ offering from question #1 above

i)……

ii)……

iii)……

Reasons for Fifth service/ offering from question #1 above

i)……

ii)……

iii)……

3a. Were you taking ARVs inside prison/ corrections before your release/

	discharge?<br>☐ 0) No<br>☐ 1) Yes<br>☐ 2) Don't know<br><br>***If Response is "0" or "2," please Skip to Question #4***<br><br>3b. Did you receive a supply of ARVs to use on the outside/ in the community before your release/ discharge?<br>☐ 0) No<br>☐ 1) Yes<br>☐ 2) Don't know<br><br>***If Response is "0" or "2," please Skip to Question #4***<br><br>3c. If you received a supply of ARVs before your most recent discharge/ release, how many days worth of ARVs did you get?<br>☐ 0) 30 days<br>☐ 1) 60 days<br>☐ 2) 90 days<br>☐ 3) 120 days<br>☐ 4) Other; please specify: ___________________________<br><br>3d. Was it enough ARVs to last you until you first went to the doctor / ART clinic in the community?<br>☐ 0) No<br>☐ 1) Yes<br><br>3e. How many days worth of ARVs do you think is the right amount to receive at prisons/ corrections discharge/ release?<br>☐ 0) 30 days<br>☐ 1) 60 days<br>☐ 2) 90 days<br>☐ 3) 120 days<br>☐ 4) Other; please specify: ___________________________
	4. Looking back based on your experiences after leaving prison/ corrections,

UIC: ☐☐-☐☐-☐☐-☐☐

First 2 letters of First Name-2 Digit birth year-First 2 letters of Surname-Birth order

	what were the biggest challenges you faced in trying to get HIV care and treatment in the community?<br><br>***[READ responses aloud and Mark <u>ALL</u> that apply]:***<br>☐ 0) Did not know where to go to get HIV treatment in the community<br>☐ 1) The ART clinic was too far away<br>☐ 2) Did not have time to go to the ART clinic<br>☐ 3) Did not have money to go to the ART clinic<br>☐ 4) The cues at the ART clinic were too long<br>☐ 5) Worried people you knew would see you at the ART clinic<br>☐ 6) Felt ashamed about having to go to the ART clinic for treatment<br>☐ 7) Health workers at the clinic treated you badly because you have HIV<br>☐ 8) Health workers treated you badly because you had been in prison/ corrections<br>☐ 9) Other; please specify:…………………………………………………………………
Domains: *Physical and financial access to services; Acceptability (for new model); Demand (for new model); Integration (for new model)*	5. Now I am going to read you a list of services and other offerings that <u>could</u> be provided in the future to help inmates as they get ready to leave prison/ corrections. If you had to go through the process of leaving prison/ corrections again, which of these would you prefer to get to help you connect with HIV care and treatment in the community?<br><br>***Mark <u>ALL</u> that apply (READ each responses aloud):***<br>☐ 0) Referral to an organization helping inmates (e.g. Chreso, PRISCCA, etc.)<br>☐ 1) Help with transportation<br>☐ 2) A basic mobile phone<br>☐ 3) Air time<br>☐ 4) Information on how to contact a company or job site looking to hire former inmates for piece work.<br>☐ 5) Information on how to contact a support group helping people with alcohol or drug problems in the community<br>☐6) Anything else not listed here that could help inmates leaving prison/ corrections get HIV treatment in the community? If so, please mention:<br>……………………………………………………………………………………………

6. ***[Read verbatim, filling in the names of the services/ offerings provided under question #5 above]:*** Please give at least one reason why you think ***[fill in 1st option from question #5 above]*** would be helpful?

[Use free listing method every time a participant gives a new reason, as follows]:
You mentioned ***[fill in reason given]*** as a reason you think ***[fill in first option from question #5 above]*** would be helpful, was there any other reason you thought ***[fill in first option]*** would be helpful?

[Continue summarizing the reasons given by the participant for the first option from question #5 above until the participant provides no more new reasons. Then proceed to the next option from question #5 above, and repeat the process].

Please give at least one reason why you think ***[fill in 2nd option from question #5 above]*** would be helpful?....

Reasons for First service/ offering from question #5 above

i)……………………………………………………………………………………………………

ii)……………………………………………………………………………………………………

iii)……………………………………………………………………………………………………

Reasons for Second service/ offering from question #5 above

i)……………………………………………………………………………………………………

ii)……………………………………………………………………………………………………

iii)……………………………………………………………………………………………………

Reasons for Third service/ offering from question #5 above

i)……………………………………………………………………………………………………

ii)……………………………………………………………………………………………………

iii)……………………………………………………………………………………………………

Reasons for Fourth service/ offering from question #5 above

i)……………………………………………………………………………………………………

	ii)……………………………………………………………………………………………………<br><br>iii)……………………………………………………………………………………………………<br><br>**Reasons for Fifth service/ offering from question #5 above**<br>i)……………………………………………………………………………………………………<br><br>ii)……………………………………………………………………………………………………<br><br>iii)……………………………………………………………………………………………………
	7. Now I am going to read you a list of people who could be made available for inmates to contact after release. If you had to go through the process of leaving prison/ corrections again, which of these people, if any, would you like to receive information on how to contact to help you get HIV treatment in the community?<br><br>***Mark <u>ALL</u> that apply (READ each responses aloud):***<br>☐ 0) someone who was in a similar situation as you, being HIV positive who successfully got HIV treatment in the community and who could be a mentor or friend to you on the outside (i.e. someone like a former inmate peer educator)<br>☐ 1) a counsellor in the community to support your mental health needs<br>☐ 2) a support group helping people living with HIV in the community<br>☐ 3) a support group helping people with alcohol or drug problems in the community<br>☐ 4) I would prefer not to receive information on how to contact any of these people
	8. ***[Read verbatim, filling in the names of the services/ offerings provided under question #7 above]:*** Please give at least one reason why you think ***<u>[fill in 1st option from question #7 above]</u>*** would be helpful?<br><br>***[Use free listing method every time a participant gives a new reason, as follows]:***<br>You mentioned ***<u>[fill in reason given]</u>*** as a reason you think ***<u>[fill in first option from question #7 above]</u>*** would be helpful, was there any other reason you thought ***<u>[fill in first option]</u>*** would be helpful?<br><br>***[Continue summarizing the reasons given by the participant for the***

first option from question #7 above until the participant provides no more new reasons. Then proceed to the next option from question #4 above, and repeat the process].

Please give at least one reason why you think ***[fill in 2nd option from question #7 above]*** would be helpful?....

Reasons for First service/ offering from question #7 above

i)……

ii)……

iii)……

Reasons for Second service/ offering from question #7 above

i)……

ii)……

iii)……

Reasons for Third service/ offering from question #7 above

i)……

ii)……

iii)……

Reasons for Fourth service/ offering from question #7 above

i)……

ii)……

iii)……

Reasons for Fifth service/ offering from question #7 above

i)……

ii)……

iii)……

UIC: ☐☐-☐☐-☐☐-☐☐

First 2 letters of First Name-2 Digit birth year-First 2 letters of Surname-Birth order

SECTION II: TRANSITIONAL CARE SERVICES (26 items, 28 questions)

[***Read verbatim:*** *"Now I am going to ask you about your preferences for services to help releasees connect to HIV care and treatment in the community. Imagine that you had to go through the release process again. Looking back based on your experience with the release process and getting HIV care and treatment in the community, what help would you want to stay healthy?"]*

Domains: *Releasee experiences with re-integration…; Acceptability (new model); Implementation (new model); Practicality (new model)*	1. Would you prefer….. <br>***Mark ONE [READ responses aloud]:*** ☐ 0) to have someone confirm an appointment day and time for your first ART Clinic visit after release ☐ 1) to just go to the clinic without an appointment day and time
Domains: *Releasee experiences with re-integration…; Acceptability (new model); Practicality (new model)*	2. Would you prefer….. <br>***Mark ONE (READ responses aloud):*** ☐ 0) to have someone accompany you to your first ART Clinic visit after release ☐ 1) to go to your first ART Clinic visit alone <br>***If 0 Skip to Question #8.*** <br>2a. Please give at least one reason why you would prefer to go to your first ART Clinic visit alone? <br>***[Use free listing method as follows]:*** You mentioned ***[fill in reason given]*** as a reason you would like to go to your first ART Clinic visit alone, was there any other reason for why you would like to go to your first ART Clinic visit alone? <br>***[Continue summarizing the reasons given by the participant for preferring to go to the first ART Clinic visit alone until the participant provides no more new reasons. Then proceed to the next question].***

	**Reasons for Preferring to Go to First ART Clinic Visit Alone**<br><br>i)………………………………………………………………………………<br>ii)………………………………………………………………………………<br>iii)………………………………………………………………………………
Domains:*Releasees experiences with re-integration…; Acceptability (new model); Practicality (new model)*	3. If you would like someone to accompany you to your first ART Clinic visit after release, would you prefer someone who…..<br><br>***Mark <u>ONE</u> (READ responses aloud):***<br>☐ 0) is living with HIV<br>☐ 1) is not living with HIV<br>☐ 2) not important
Domains: *Releasee experiences with re-integration…; Acceptability (new model); Practicality (new model)*	4. If you would like someone to accompany you to your first ART Clinic visit after release, would you prefer…..<br><br>***Mark <u>ONE</u> (READ responses aloud):***<br>☐ 0) A man<br>☐ 1) A woman<br>☐ 2) not important
Domains: *Releasee experiences with re-integration…; Acceptability (new model); Practicality (new model)*	5. If you would like someone to accompany you to your first ART Clinic visit after release, would you prefer someone…..<br><br>***Mark <u>ONE</u> (READ responses aloud):***<br>☐ 0) who is a former inmate<br>☐ 1) who is not a former inmate<br>☐ 2) not important

***Domains:** Releasee experiences with re-integration…; Acceptability (new model); Practicality (new model)*	6. If you would like someone to accompany you to your first ART Clinic visit after release, would you prefer someone…<br><br>***Mark <u>ONE</u> (READ responses aloud):***<br>☐ 0) who is older than you<br>☐ 1) who is younger than you<br>☐ 2) who is the same age as you<br>☐ 3) not important
***Domains:** Releasee experiences with re-integration…; Acceptability (new model); Practicality (new model)*	7. If you would like someone to accompany you to your first ART Clinic visit after release, would you prefer someone...<br><br>***Mark <u>ONE</u> (READ responses aloud):***<br>☐ 0) who knows you<br>☐ 1) who doesn't know you<br>☐ 2) not important
***Domains:** Demand (for new model); Implementation; practicality; adaption; Expansion*	8. Let's say that you were able to find HIV care and treatment in your community after release. For continuing to get your HIV treatment in the community, would you prefer ….<br><br>***Mark <u>ONE</u> (READ responses aloud):***<br>☐ 0) to go to the ART clinic in the community<br>☐ 1) to go to the ART clinic in the community <u>but during special "off" hours when the clinic isn't as busy.</u><br>☐ 2) to go to a clinic run by a non-governmental group in the community like a CBO (e.g. Chreso) or a church<br>☐ 3) to go to the nearest corrections facility / prison clinic<br>☐ 4) other; please specify: …………………………………………………
***Domains:** Physical and faincncial access to services; Acceptability;*	9. If you had to choose from the following list, what 3 things would you consider the <u>most important in deciding where</u> to receive HIV care and treatment in your community after release?<br><br>***Mark <u>the top three choices</u> (READ responses aloud):***<br>☐ 0) Privacy and confidentiality<br>☐ 1) Transportation support to travel to the clinic<br>☐ 2) A clinic that is close or easy to get to

	☐ 3) Social support to help you take your ARVs the right way every day<br>☐ 4) Availability of other health services at the same clinic (like treatment for sexually transmitted diseases or family planning)<br>☐ 5) Convenient hours at the clinic (like being open in the late afternoon or early evening)<br>☐ 6) Not being judged about having HIV by health workers at the clinic<br>☐ 7) Not being judged about being a former inmate/ ex-offender by health workers at the clinic<br>☐ 8) Other; please explain: ……………………………………………………<br>…………………………………………………………………………………………
Domains: *Acceptability; Demand; Implementation; practicality; adaption; intrgration*	10. Let's say that you were able to get HIV care and treatment in your community after release. For your visits to the ART clinic, <u>in addition to</u> seeing the doctor or nurse, would you prefer to talk to someone who was in a similar situation like you during your visit about challenges you're facing with your health or just see the doctor or nurse for ART and go back home?<br><br>***Mark <u>ONE</u> (READ responses aloud):***<br>☐ 0) See the doctor or nurse for ART and go back home<br>☐ 1) <u>In addition to</u> seeing the doctor or nurse, talk to a trained peer who was in a similar situation as you, being <u>HIV positive ***AND*** a former inmate</u> who now is getting HIV treatment and doing well in the community<br>☐ 2) <u>In addition to</u> seeing the doctor or nurse, talk to a trained peer who is <u>HIV positive but **NOT** a former inmate</u> who is getting HIV treatment and doing well in the community<br>☐ 3) <u>In addition to</u> seeing the doctor or nurse, talk to a trained counsellor to support your mental health needs
**Domains:** Acceptability; Demand;	11. Let's say that you were able to get HIV care and treatment in your community after release. <u>For getting refills of your ARVs</u>, if you had to choose <u>one option</u> would you prefer ….

	Mark ONE (READ responses aloud):<br>☐ 0) to collect your ARVs from your clinic<br>☐ 1) to collect your ARVs from a place outside the clinic (such as a health post, school, church, or another meeting place in the community)<br>☐ 2) Other; please explain:<br>……………………………………………………………………………………………<br><br>***If Response is "1" or "2," please Skip to Question #13***
Domains: *Acceptability; Demand; implementation; practicality; adaption; integration; expansion*	12. Let's say that you were able to get HIV care and treatment in your community after leaving prison/ corrections. Now I am going to ask you to think about different options for collecting refills of your ARVs at the clinic. Please let me know the top 3 ways you would prefer to collect ARVs at the clinic...<br><br>***Mark the top three choices (READ responses aloud):***<br>☐ 0) to collect your ARVs from the pharmacist at the clinic<br>☐ 1) to collect your ARVs from a nurse at the clinic<br>☐ 2) to collect your ARVs from a doctor at the clinic<br>☐ 3) to collect your ARVs from a counsellor at the clinic<br>☐ 4) to collect your ARVs from a peer treatment supporter at the clinic<br>☐ 5) to collect your ARVs from a peer support group at the clinic<br>☐ 6) to collect your ARVs at the clinic in another way not mentioned; (if so, please explain……………………………………….<br>……………………………………………………………………………………
Domains: *Acceptability; Demand; implementation; practicality; Adaption; Integration; Expansion*	13. Let's say that you were able to get HIV care and treatment in your community after leaving prisons/ corrections. Now I am going to ask you to think about different options for collecting refills of your ARVs outside the clinic. Please let me know the top 3 ways you would prefer to collect ARVs outside the clinic...<br><br>***Mark ALL that apply (READ responses aloud):***

	☐ 0) to collect your ARVs from a pharmacist <u>outside the clinic</u> in a safe and private space in the community<br>☐ 1) to collect your ARVs from a nurse <u>outside the clinic</u> in a safe and private space in the community<br>☐ 2) to collect your ARVs from a counsellor <u>outside the clinic</u> in a safe and private space in the community<br>☐ 3) to collect your ARVs from a peer educator <u>outside the clinic</u> in a safe and private space in the community<br>☐ 4) to collect your ARVs from a peer support group <u>outside the clinic</u> in a safe and private space in the community<br>☐ 5) to collect your ARVs <u>outside the clinic</u> in another way not listed here. If so, please explain……………………………….<br>…………………………………………………………………………………..
Domains: *Releasee experiences of re-integration…; acceptability; demand; implementation; adaption; integration*	14. If a support group made up of other patients were available for you to talk about your problems, would you be…..<br><br>***Mark <u>ONE (READ responses aloud)</u>:***<br>☐ 0) willing to join<br>☐ 1) not willing to join<br><br>***If Response is "0," please Skip to Question #15***<br><br>14a. Please give at least one reason why you would prefer not to join a support group made up of other patients?<br><br>***[Use free listing method as follows]:***<br>You mentioned ***<u>[fill in reason given]</u>*** as a reason you prefer not to join a support group, was there any other reason why you would prefer not to join a support group?<br><br>***[Continue summarizing the reasons given by the participant until the participant provides no more new reasons. Then proceed to the next question].***<br><br>**Reasons for Not to Join a Support Group**<br><br>i)……………………………………………………………………………………<br><br>ii)……………………………………………………………………………………

	iii)…………………………………………………………………………………………
**Domain:** *acceptability; practicality; adaption; integration*	15. If you would be willing to join a support group, what kinds of help or services would you want to receive from the group? Please choose all that apply.<br><br>***Mark ALL that apply (READ responses aloud):***<br>☐ 0) Refills of ARVs<br>☐ 1) Adherence counselling<br>☐ 2) Information about positive living and how to stay healthy<br>☐ 3) Group counselling done by a trained mental health counsellor on how to cut down on using alcohol<br>☐ 4) Group counselling done by a trained mental health counsellor on how to cut down on using drugs<br>☐ 5) Referrals to support services in the community<br>☐ 6) Vital sign checks<br>☐ 7) Screening for tuberculosis (i.e. "TB")<br>☐ 8) Other (*please specify*):<br>____
**Domains:** *acceptability*	16. If you would be willing to join a support group, would you prefer that other group members….<br><br>***Mark ONE (READ responses aloud):***<br>☐ 0) were older than you<br>☐ 1) were younger than you<br>☐ 2) were about the same age as you<br>☐ 3) not important
**Domain**	17. If you would be willing to join a support group, would you

	prefer that other group members….<br><br>***Mark <u>ONE</u> (READ responses aloud):***<br>☐ 0) were men<br>☐ 1) were women<br>☐ 2) not important
	18. If you would be willing to join a support group, how many people total, including yourself, would you prefer to have in the group….<br><br>***Mark <u>ONE</u> (READ responses aloud):***<br>☐ 0) 2 to 5 people<br>☐ 1) 6 to 10 people<br>☐ 2) 11 to 20 people<br>☐ 3) more than 20 people<br>☐ 4) not important
Domains: *acceptability*	19. If you would be willing to join a support group, would you prefer that other group members….<br><br>***Mark <u>ONE</u> (READ responses aloud):***<br>☐ 0) were former inmates/ ex-offenders<br>☐ 1) were not former inmates/ ex-offenders<br>☐ 2) not important
	20. If you would be willing to join a support group, would you prefer that a nurse, counselor or other health worker assigned members to different groups, or that members picked their own groups….<br><br>***Mark <u>ONE</u> (READ responses aloud):***<br>☐ 0) groups assigned by a nurse, counselor, or other health worker<br>☐ 1) members pick their own groups<br>☐ 2) not important
Domains: *acceptability*	21. If you would be willing to join a support group, would you prefer that the group was led by a trained person who was….<br><br>***Mark <u>ONE</u> (READ responses aloud):***<br>☐ 0) a psychosocial counselor, meaning someone with training on how to help people with mental health problems

	like depression and problems using alcohol or drugs<br>☐ 1) a peer health educator or treatment supporter from the community<br>☐ 2) a former inmate peer health educator<br>☐ 3) a nurse<br>☐ 4) a clinician/ doctor<br>☐ 5) not important<br>☐ 6) Other; if so please explain……………………………………………
Domains: *Acceptability; Implementation; practicality; adaption; integration*	22. If you would be willing to join a support group, where would you prefer that the group met….<br><br>***Mark <u>ONE</u> (READ responses aloud):***<br>☐ 0) at the ART clinic<br>☐ 1) at the health center but in a space different than the ART clinic<br>☐ 2) at a church<br>☐ 3) at a community-based organization<br>☐ 4) at a school<br>☐ 5) at a community hall or centre<br>☐ 6) at a health post in the community<br>☐ 7) not important<br>☐ 8) Other; if so please explain……………………………………………
Domains: *acceptability*	23. If you would be willing to join a support group, how often would you prefer that the group met….<br><br>***Mark <u>ONE</u> (READ responses aloud):***<br>☐ 0) once a week<br>☐ 1) once every 2 weeks (i.e. fortnightly)<br>☐ 2) once per month<br>☐ 3) once every 2 months<br>☐ 4) once every 3 months<br>☐ 5) once every 6 months<br>☐ 6) Other; if so please explain……………………………………………
Domains: *acceptability*	24. If you would be willing to join a support group, on which day of the week, Monday through Sunday, would you prefer to come for your support group meeting…..<br><br>***Mark <u>ONE</u> (do NOT read to respondent):***<br>☐ 0) Monday<br>☐ 1) Tuesday

	☐ 2) Wednesday<br>☐ 3) Thursday<br>☐ 4) Friday<br>☐ 5) Saturday<br>☐ 6) Sunday<br>☐ 7) Not important
Domains: *acceptability*	25. If you would be willing to join a support group, at what times of the day would you be willing/able to come to support group meetings…..<br><br>***Mark ALL that apply (do NOT read to respondent):***<br>☐ 0) 0600 – 0800<br>☐ 1) 0800 – 1000<br>☐ 2) 1000 – 1200<br>☐ 3) 1200 – 1400<br>☐ 4) 1400 – 1600<br>☐ 5) 1600 – 1800<br>☐ 6) 1800 – 2000<br>☐ 7) 2000 – 2400<br>☐ 8) Not important
	26. *If you would be willing to join a support group, what sorts of help would you need to come to support group meetings…..*<br><br>***Mark ALL that apply (READ responses aloud):***<br>☐ 0) Transportation assistance<br>☐ 1) Child care/ helping watching your young children<br>☐ 2) A SMS/ text message reminder about the time and location of the meeting<br>☐ 3) A peer or other friend or mentor to accompany you to the meeting<br>☐ 4) Information on how to explain the purpose of the meeting to your partner or family<br>☐ 5) I would not need any help to come to the support group meetings<br>☐ 6) Other; please explain:…………………………………………………….
